# Supplementary material for: Linkage Disequilibrium and Inversion-Typing of the Drosophila melanogaster Genome Reference Panel
Source: G3 (Bethesda). 2015 Jun 10;5(8):1695–701. doi: 10.1534/g3.115.019554 (PMC4528326; doi:10.1534/g3.115.019554)
Supplement: Supporting Information [file supp_5_8_1695__index.html]

Linkage Disequilibrium and Inversion-Typing of the Drosophila melanogaster Genome Reference Panel — Supporting Information 

# Linkage Disequilibrium and Inversion-Typing of the *Drosophila melanogaster* Genome Reference Panel

## Supporting Information for Houle and Márquez, 2015

**Files in this Data Supplement:**

- Supporting Information - Figure S1, descriptions of Files S1-S3, Table S1, and Literature Cited (PDF, 217 KB)
- Figure S1 - Mean number of sites correlated with variant sites at r2>0.5 as a function of minor allele frequency for sites within and outside common inversions. (PDF, 141 KB)
- Table S1 - Inferred kilobase pairs of African ancestry in homozygous inverted regions. (PDF, 83 KB)
