# Supplementary material for: Linkage Disequilibrium and Inversion-Typing of the Drosophila melanogaster Genome Reference Panel
Source: G3 (Bethesda). 2015 Jun 10;5(8):1695–701. doi: 10.1534/g3.115.019554 (PMC4528326; doi:10.1534/g3.115.019554)
Supplement: Supporting Information [file supp_g3.115.019554_019554SI.pdf]

G3 · Genes | Genomes | Genetics

Linkage Disequilibrium and Inversion-Typing of the *Drosophila melanogaster* Genome Reference Panel

David Houle[*,1]
Eladio J. Márquez[*,2]
Department of Biological Science
Florida State University
Tallahassee, FL 32308

*Department of Biological Science, Florida State University, Tallahassee, FL 32306-4295, USA.

[1]Corresponding author: Department of Biological Science, Florida State University, Tallahassee, FL 32306-4295, USA. E-mail: dhoule@bio.fsu.edu

[2] Current address: The Jackson Laboratory for Genomic Medicine, Farmington, CT 06030, USA

DOI: 10.1534/g3.115.019554

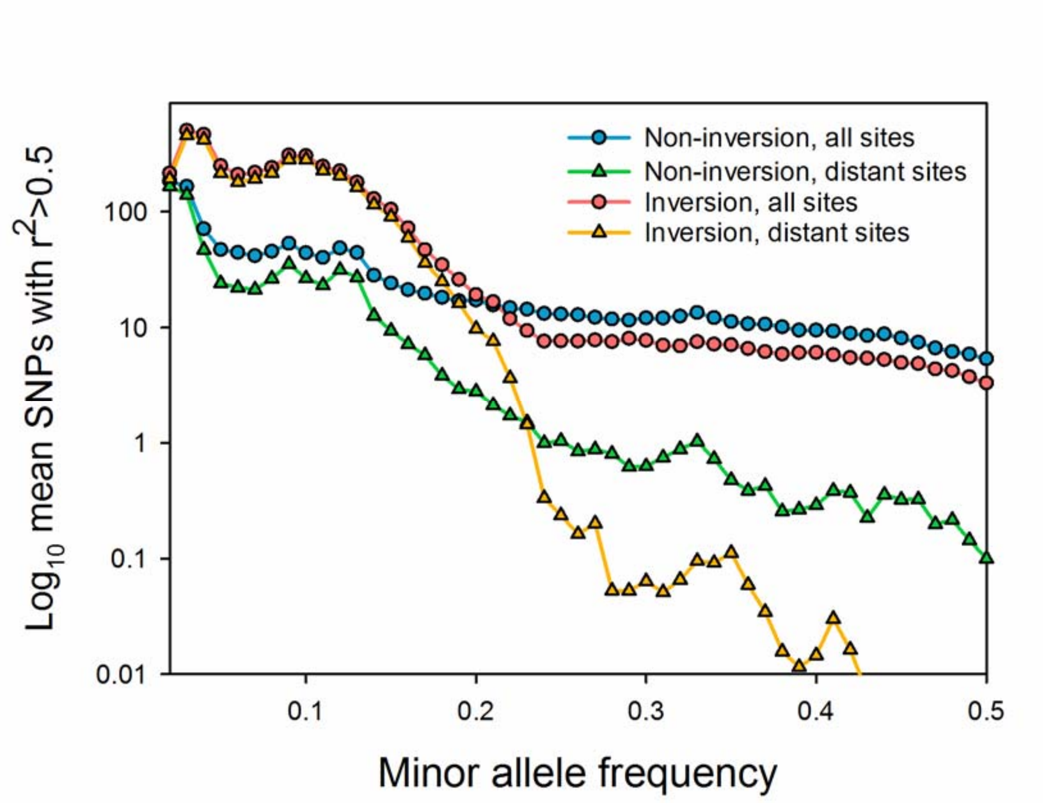

Figure S1 Mean number of sites correlated with variant sites at $r^2>0.5$ as a function of minor allele frequency for sites within and outside common inversions. We treated the distal segment of chromosome 3R as part of In(3R)Mo (Corbett-Detig and Hartl 2012).

Supporting Files

All supporting files are archived and available at http://dx.doi.org/10.5061/dryad.06jt7

File S1

HouleMarquezSASfiles.zip . Zip file with two SAS programs and example data set. Gcorrlimits.sas is a stand-alone program that demonstrates how we calculated the limits on which allele frequencies can be correlated at $r^2 \geq 0.5$. CalcHighCorr.sas reads the SAS data set gcorrexampledata.sas7bdat to demonstrate the calculation of correlations as a measure of linkage disequilibrium. Programs are written in SAS 9.3 (SAS Institute 2011).

File S2

LD205results.zip. Correlations of all SNP pairs with $r^2 \geq 0.5$ in the Freeze2 of the DGRP. Zip file contains separate csv files for each chromosome arm.

File S3

HouleMarquezF3_PCscores.csv. Supporting Table: Inversion-typing of DGRP lines for the three common inversions In(2L)t, In(2R)NS, and In(3R)Mo, and heterozygosity of chromosome regions.

Table S1 Inferred kilobase pairs of African ancestry in homozygous inverted regions.

		Consensus*		Mismatch†	
Chromosome	Predicted Karyotype	Mean ± S.D.	N	Mean ± S.D.	N
2L	Standard	2,022 ±1,081	161	--	
	In(2L)t	13,605±2,494	19	14,713 ± 1,098	4
2R	Standard	412 ±194	161	5,840	1
	In(2R)NS	6,385±3,057	7	874	1
3R	Standard	1,504 ±3,459	175	1,051± 466	2
	In(3R)MO	80 ± 67	16	64 ± 40	4

* Consensus lines are assigned the same inversion type by our LD-based PC classification and by previous studies (Corbett-Detig and Hartl 2012; Huang et al. 2012).
† Mismatch lines are assigned an LD PC classification that disagrees with one of the previous studies.

Literature Cited

Corbett-Detig, R.B., and D.L. Hartl, 2012 Population Genomics of Inversion Polymorphisms in Drosophila melanogaster. PLoS Genetics 8: e1003056.

Huang, W., S. Richards, M.A. Carbone, D. Zhu, R.R.H. Anholt *et al.*, 2012 Epistasis dominates the genetic architecture of Drosophila quantitative traits. Proceedings of the National Academy of Sciences of the United States of America 109: 15553-15559.

SAS Institute, Inc., 2011 The SAS System for Windows, Release 9.3. SAS Institute, Cary, NC.
