# Supplementary material for: Linkage Disequilibrium and Inversion-Typing of the Drosophila melanogaster Genome Reference Panel
Source: G3 (Bethesda). 2015 Jun 10;5(8):1695–701. doi: 10.1534/g3.115.019554 (PMC4528326; doi:10.1534/g3.115.019554)
Supplement: Supporting Information [file supp_g3.115.019554_FigureS1.pdf]

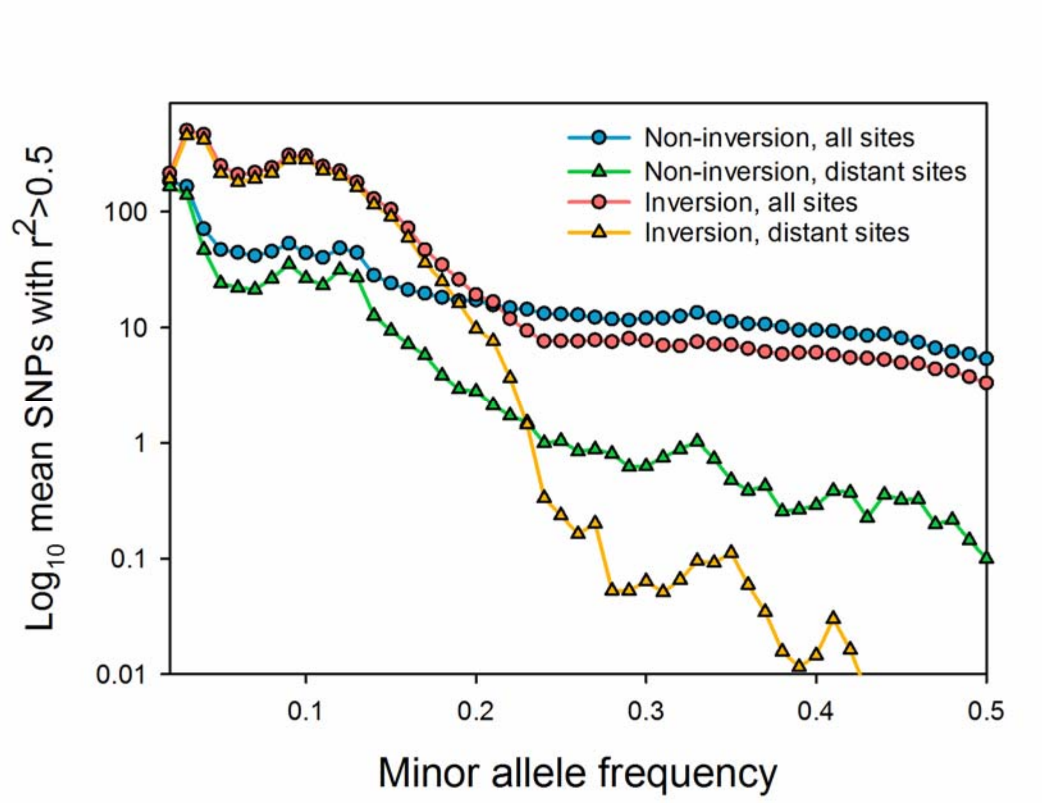

Figure S1 Mean number of sites correlated with variant sites at r^2>0.5 as a function of minor allele frequency for sites within and outside common inversions. We treated the distal segment of chromosome 3R as part of In(3R)Mo (Corbett-Detig and Hartl 2012).
