# Supplementary material for: Linkage Disequilibrium and Inversion-Typing of the Drosophila melanogaster Genome Reference Panel
Source: G3 (Bethesda). 2015 Jun 10;5(8):1695–701. doi: 10.1534/g3.115.019554 (PMC4528326; doi:10.1534/g3.115.019554)
Supplement: Supporting Information [file supp_g3.115.019554_TableS1.pdf]

Table S1 Inferred kilobase pairs of African ancestry in homozygous inverted regions.

Chromosome	Predicted Karyotype	Consensus*		Mismatch†	
		Mean ± S.D.	N	Mean ± S.D.	N
2L	Standard	2,022 ±1,081	161	--	
	In(2L)t	13,605±2,494	19	14,713 ± 1,098	4
2R	Standard	412 ±194	161	5,840	1
	In(2R)NS	6,385±3,057	7	874	1
3R	Standard	1,504 ±3,459	175	1,051± 466	2
	In(3R)MO	80 ± 67	16	64 ± 40	4

* Consensus lines are assigned the same inversion type by our LD-based PC classification and by previous studies (Corbett-Detig and Hartl 2012; Huang et al. 2012).

† Mismatch lines are assigned an LD PC classification that disagrees with one of the previous studies.
